# Tunable Contact Types and Interfacial Electronic Properties in TaS_2_/MoS_2_ and TaS_2_/WSe_2_ Heterostructures

**DOI:** 10.3390/molecules28145607

**Published:** 2023-07-24

**Authors:** Xiangjiu Zhu, Hongxing Jiang, Yukai Zhang, Dandan Wang, Lin Fan, Yanli Chen, Xin Qu, Lihua Yang, Yang Liu

**Affiliations:** Key Laboratory of Functional Materials Physics and Chemistry of the Ministry of Education, Key Laboratory of Preparation and Application of Environmental Friendly Materials, College of Physics, Jilin Normal University, Changchun 130103, China; zxj1474131433@126.com (X.Z.);

**Keywords:** two-dimensional heterostructures, first-principles calculations, electronic properties, electrical contact

## Abstract

Following the successful experimental synthesis of single-layer metallic 1T-TaS_2_ and semiconducting 2H-MoS_2_, 2H-WSe_2_, we perform a first-principles study to investigate the electronic and interfacial features of metal/semiconductor 1T-TaS_2_/2H-MoS_2_ and 1T-TaS_2_/2H-WSe_2_ van der Waals heterostructures (vdWHs) contact. We show that 1T-TaS_2_/2H-MoS_2_ and 1T-TaS_2_/2H-WSe_2_ form n-type Schottky contact (n-ShC type) and p-type Schottky contact (p-ShC type) with ultralow Schottky barrier height (SBH), respectively. This indicates that 1T-TaS_2_ can be considered as an effective metal contact with high charge injection efficiency for 2H-MoS_2_, 2H-WSe_2_ semiconductors. In addition, the electronic structure and interfacial properties of 1T-TaS_2_/2H-MoS_2_ and 1T-TaS_2_/2H-WSe_2_ van der Waals heterostructures can be transformed from n-type to p-type Schottky contact through the effect of layer spacing and the electric field. At the same time, the transition from Schottky contact to Ohmic contact can also occur by relying on the electric field and different interlayer spacing. Our results may provide a new approach for photoelectric application design based on metal/semiconductor 1T-TaS_2_/2H-MoS_2_ and 1T-TaS_2_/2H-WSe_2_ van der Waals heterostructures.

## 1. Introduction

Due to their remarkable material properties and enormous potential for use in a wide variety of technological device applications, novel 2D materials have attracted a significant amount of attention from researchers. Graphene [1,2] as the first material to promote the field of 2D materials research has many remarkable properties, such as massless Dirac fermions [3], high carrier mobility [4], high conductivity [5], and an unconventional quantum Hall effect at room temperature [6]. However, high-speed electrical applications like field-effect transistors are limited by the lack of a bandgap in graphene [7]. Therefore, the research community has been actively searching for 2D semiconductor materials with excellent properties and applications. In recent years, TMDs, a whole new class of 2D materials, have been studied as the most attractive materials due to their excellent properties. Because of their inherent advantages, such as a high surface-to-volume ratio, no dangling bonds, and excellent carrier mobility, TMDs [8,9,10,11] are important technical materials for various energy, electronic, and optoelectronic applications in the future.

Among TMDs, 2H-MoS_2_ and 2H-WSe_2_, the two most typical TMDs materials, have fascinating properties and a wide range of applications. The bulk structure of MoS_2_ is stratified, with weak van der Waals (vdW) forces between layers. Unlike graphene, monolayer MoS_2_ is a direct bandgap [7,8] semiconductor with a bandgap of 1.8 eV. Moreover, single-layer MoS_2_ has a high on/off current ratio of about 10^8^ and a high carrier mobility [9] of 200 cm^2^/V^−1^s^−1^ at room temperature, making it promising in field-effect transistors [7] (FETs), photodetectors [10], and electroluminescent devices, thus demonstrating its considerable application potential. So far, the structural, mechanical, electronic, and transport properties of MoS_2_ monolayers have been extensively studied, both experimentally and theoretically [11,12,13,14,15,16,17]. The results show that the physical properties of MoS_2_ monomolecular film are very sensitive to external conditions, such as the strain and electric field. Furthermore, 2D WSe_2_ is an indirect bandgap semiconductor with a rather large bandgap. In the past few years, the electronic properties, thermoelectric response [18], and strain engineering [11,19,20,21] of WSe_2_ have been extensively studied. At the same time, important applications of WSe_2_ in transistors [22,23,24], phototransistors [25], circuits [26,27], and magnesium-ion batteries [28] have also been discussed. Furthermore, single-layer and few-layer WSe_2_ have been experimentally synthesized [29,30,31], which makes the fabrication of high-performance WSe_2_-based nanoelectronics devices promising. Different from 2D MoS_2_ and WSe_2_ monolayers, we note that 1T-TaS_2_, as an emerging 2D layered TMD, is one of the most studied TMDs due to its unexpected physical properties. The structure of 1T-TaS_2_ is similar to the 2H phase of MoS_2_, but it exhibits different metallic properties [32,33], so it is very important for our next research work. 

The electrical contacts between metals and semiconductors are frequently used in modern electronic and optoelectronic devices, which can not only greatly improve the charge injection efficiency of semiconductors, but also improve the performance of electronic devices. The study of metal/semiconductor interfacial contacts is a crucial step in the construction of energy-efficient and high-performance electronic devices. The functionality of the device may be compromised partially or totally in the event of inappropriate contact between the metal and the semiconductor. Therefore, the formation of a low or eliminated Schottky barrier height (SBH) [22,34] in the metal-semiconductor junction (MSJ) from the Schottky to the ohmic contact is critical for the fabrication of high-performance nanodevices. Since most 2D metal-semiconductor interfaces are essentially Schottky interfaces [35,36], there will be intrinsic and extrinsic limitations, including surface defects, work function mismatch, and sustainable doping strategies; thus, Schottky Converting base contact to ohmic contact is indeed a challenging task.

The high contact resistance [37] of transition metal dichalcogenide (TMDs) devices is one of the bottlenecks limiting the application of TMDs in various fields. The contact [38] resistance of TMD-based devices is closely related to the contacted metal/TMDs interface and band alignment [39]. So far, a large number of theoretical experiments [40] have demonstrated that the vdW interaction can generate good contact properties in metal-transition metals [41,42] and semimetals-transition metals [43]. Therefore, vdW contacts have a very wide range of applications.

To date, the tunable Schottky barrier and electronic properties of the 1T-TaS_2_/2H-MoS_2_ (2H-WSe_2_) combination have not yet been investigated. Based on this idea, we constructed 1T-TaS_2_, 2H-MoS_2_ (2H-WSe_2_) monolayer structures, respectively. In this work, we performed first-principles calculations to investigate the atomic and electronic structures of 1T-TaS_2_/2H-MoS_2_ (2H-WSe_2_) vdWHs and their tunable electronic structures under interlayer spacing and the electric field. The vdW interaction between metallic 1T-TaS_2_ and semiconducting 2H-MoS_2_ (2H-WSe_2_) monolayers makes the heterostructure energetically feasible and preserves the intrinsic properties of the two constituent monolayers. Our results predict that 1T-TaS_2_/2H-MoS_2_ (2H-WSe_2_) vdWHs have a tunable Schottky barrier height (SBH), and that the electronic structure and interfacial properties of 1T-TaS_2_/2H-MoS_2_ (2H-WSe_2_) vdWHs can be transformed from n-type to p-type ShC through the effect of layer spacing and the electric field. At the same time, the transition from ShC to OhC can also occur by relying on the electric field and different interlayer spacing. Our results reveal the potential role of metallic 1T-TaS_2_ as an effective metal contact to semiconductor 2H-MoS_2_ (2H-WSe_2_).

## 2. Results and Discussion

### 2.1. Geometric Structures and Electronic Properties

**Figure 1:**
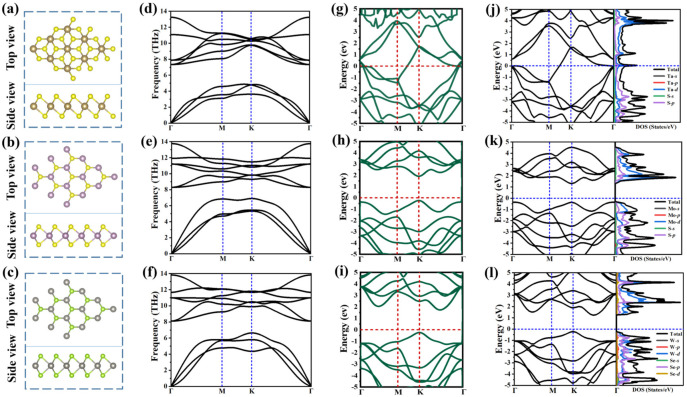
(**a**−**c**) show the optimized atomic structure (top view and side view), (**d**−**f**) phonon dispersion curve, (**g**−**i**) HSE06 projected band structures and state density of 1T-TaS_2_, 2H-MoS_2_, 2H -WSe_2_, respectively. (**j**−**l**) PBE projected band structures and state density of 1T-TaS_2_, 2H-MoS_2_, 2H -WSe_2_, respectively. The brown, yellow, purple, silver, and green balls represent tantalum, sulfur, molybdenum, tungsten, and selenium atoms, respectively.

In Figure 1, we showed the atomic structure, phonon spectrum, projected band structure, and state density of metallic 1T-TaS_2_ and semiconducting 2H-MoS_2_, 2H-WSe_2_ monolayer. After geometric optimization, the monolayers 1T-TaS_2_, 2H-MoS_2_, and 2H-WSe_2_ all show layered atomic structures, and their lattice constants are 3.186 Å, 3.184 Å, and 3.184 Å, respectively, which is consistent with previous experimental and theoretical measurements. In Figure 1a–c, it can be seen that the Ta atom in metal TaS_2_ is sandwiched between two S atoms, while the Mo atom and W atom are sandwiched between two S atoms and two Se atoms in semiconductor MoS_2_ and WSe_2_ monolayers, respectively. In addition, the 1T-TaS_2_ layer in Figure 1g shows metallic behavior, while the 2H-MoS_2_ and 2H-WSe_2_ monolayers in Figure 1h, ishow semiconductor characteristics. 2H-MoS_2_ shows a direct bandgap semiconductor while 2H-WSe_2_ shows an indirect bandgap semiconductor. The bandgap values calculated by HSE06 are 2.32 eV and 1.97 eV, and those calculated by PBE are 1.66 eV and 1.46 eV, respectively. The results show that they are close to the experimental measurements [11] of 1.80 eV and 1.65 eV, which confirms the reliability of our calculation. In general, traditional PBE methods often underestimate the bandgap of 2D semiconductors, and HSE06 can be used to predict more accurate bandgap values. However, the PBE bandgap of the 2H-MoS_2_ and 2H-WSe_2_ monolayers is closer to the experimental bandgap than the HSE06 method. Therefore, we use the PBE method for all of the following calculations. Moreover, in Figure 1h,i, it is found that, in both PBE and HSE06 functional, the CBM and VBM of 2H-MoS_2_ monolayer are located at the K point, while the CBM and VBM of 2H-WSe_2_ monolayer are not at the same high-symmetry path. In addition, the state density of 1T-TaS_2_ is shown in Figure 1g. For metal 1T-TaS_2_, the major contribution is the *d* orbital of Ta. Meanwhile, for the 2H-MoS_2_ monolayer, the CBM is dominated by the *d* orbital contribution of Mo, and the VBM is dominated by the *p* orbital contribution of S. For the 2H-WSe_2_ monolayer, the CBM is dominated by the *d* orbital contribution of W, and the VBM is dominated by the *p* orbital contribution of Se. The phonon spectrum of 1T-TaS_2_,2H-MoS_2_ and 2H-WSe_2_ are reflected in Figure 1d–f. It can be seen that the frequencies of the three considered monolayers are all positive, and there is no negative frequency at the Γ point, thus confirming their dynamic stability.

### 2.2. Structures and Electronic Properties of Heterostructures

**Figure 2:**
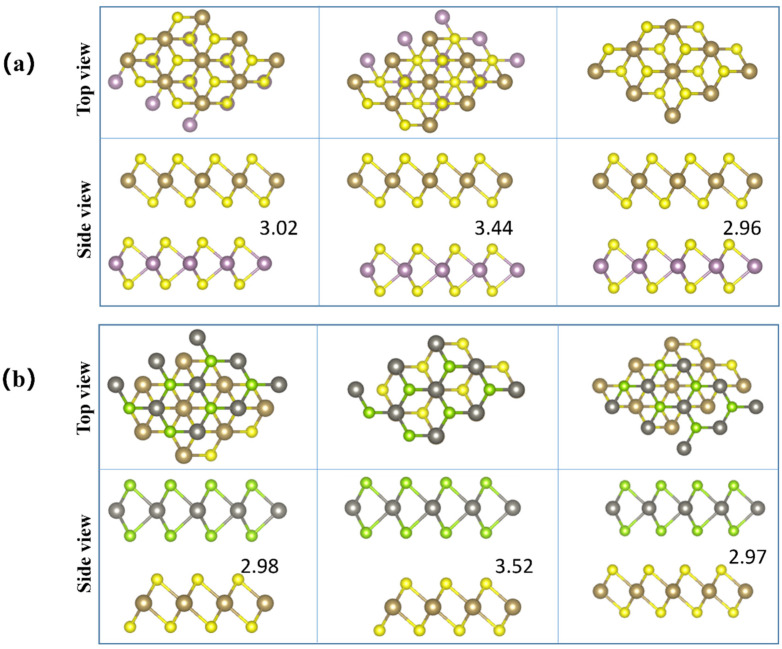
(**a**,**b**) Three different stacking forms of TaS_2_/MoS_2_, TaS_2_/WSe_2_ vdWHs, respectively. The brown, yellow, purple, silver, and green balls represent tantalum, sulfur, molybdenum, tungsten, and selenium atoms, respectively.

We constructed vdWHs by stacking the monolayers 2H-MoS_2_ and 2H-WSe_2_ in the 1T-TaS_2_ monolayer along the z direction and setting the initial equilibrium layer spacing D as 3.02 Å and 2.98 Å, which are greater than the sum of the covalent radii between Mo and S atoms and W and Se atoms, respectively. This confirmed that no covalent bond has been formed between the two constituent monolayers. It is clear that these calculated interlayer distances are comparable to other previously reported interlayer distances in vdWHs, including graphene/MoS_2_ [44] and graphene/WSe_2_ [45], which are typical vdW interactions. This finding shows that there are no chemical bonds in the 1T-TaS_2_/2H-MoS_2_(2H-WSe_2_) vdWHs (in the following, the TaS_2_/MoS_2_(WSe_2_) vdWHs stand for the 1T-TaS_2_/2H-MoS_2_(2H-WSe_2_) vdWHs). At the same time, we consider the possible stacking configurations that form these two heterogeneous structures, corresponding to (a) and (b) in Figure 2, respectively. According to the calculation results, the energy of the first diagram on the left of the two vdWHs’ stacking configurations is the lowest, and the Eb is −45.95920 eV and −45.86945 eV, respectively. Therefore, we used this stacking method to construct unit cells from (1 × 1) TaS_2_ and (1 × 1) MoS_2_, (1 × 1) TaS_2_ and (1 × 1) WSe_2_ cells, respectively. According to the formula: m − n/m + n < 5% (m, n are the lattice constants of TaS_2_ and MoS_2_(WSe_2_), respectively), the calculated lattice constants of TaS_2_/MoS_2_, TaS_2_/WSe_2_ vdWHs are both 3.18674 Å, and the lattice mismatch rate is 0.04%.

Furthermore, to verify the stability of the structure, the binding energy was calculated as *E_b_* = *E_vdW_* − *E_TaS__2_* − *E_MoS__2_* (*E_WSe__2_*), where *E_vdW_*, *E_TaS__2_*, and *E_MoS__2_* (*E_WSe__2_*) represent the total energy of the corresponding vdWHs, TaS_2_, and MoS_2_(WSe_2_) monolayers, respectively. The binding energies of TaS_2_/MoS_2_(WSe_2_) were −0.28 eV and −0.32 eV, respectively. The minus sign (“−” symbol) in the binding energy indicates that these vdWHs are energy stable. To evaluate the mechanical stability, we also calculated the elastic constant of TaS_2_/MoS_2_(WSe_2_) vdWHs. The elastic constants C_11_, C_12_, and C_66_ = (C_11_ − C_12_)/2 of TaS_2_/MoS_2_ vdWHs were calculated as 254 N/m, 63 N/m, and 95 N/m, respectively. Meanwhile, the elastic constants C11, C12, and C_66_ = (C_11_ – C_12_)/2 of TaS_2_/Wse_2_ were calculated as 294 N/m, 45 N/m, and 124 N/m, respectively. It can be found that the elastic constants C11 > C12 and C66 > 0 of vdWHs satisfy the Born-Huang criterion [46,47], indicating that vdWHs are stable. In addition, we calculate Young’s modulus and Poisson’s ratio of Y = (C_11_^2^ − C_12_^2^)/C_11_, *V* = C_12/_C_11_ and other systems. The polar graphs of Young’s modulus and Poisson’s ratio of vdWHs are described in Figure S1, supporting the information. The average Young’s modulus of TaS_2_/MoS_2_ vdWHs is 238 N/m and the average Poisson’s ratio is 0.25, while the average Young’s modulus of TaS_2_/WSe_2_ vdWHs is 287 N/m and the average Poisson’s ratio is 0.15, which are lower than graphene54. It was shown that two vdWHs are susceptible to strain regulation.

**Figure 3:**
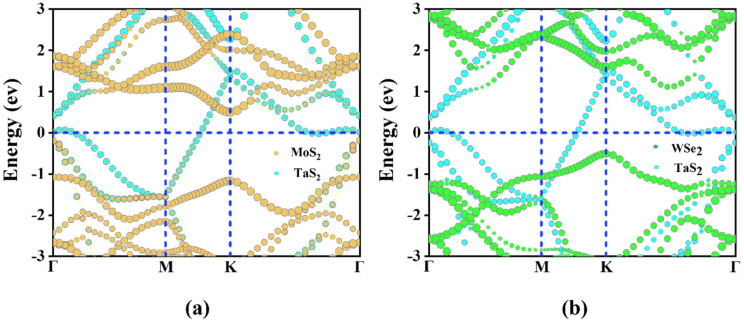
(**a**,**b**) Weighted projected band structures of TaS_2_/MoS_2_, TaS_2_/WSe_2_ vdWHs obtained by PBE calculations, respectively. The blue, yellow, and green lines represent TaS_2_, MoS_2_, WSe_2_, respectively.

**Figure 4:**
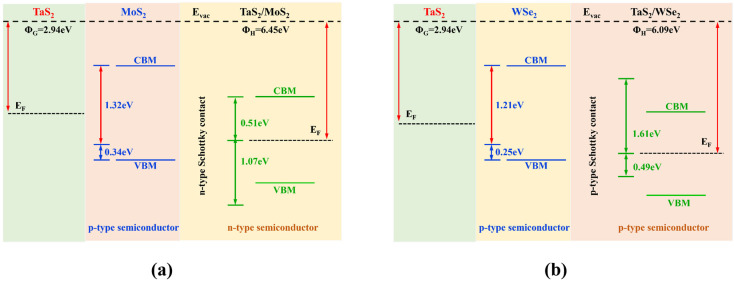
(**a**,**b**) The work functions of TaS_2_, MoS_2_, WSe_2_ monolayer and their vdWHs.

The band structure of TaS_2_/MoS_2_, TaS_2_/WSe_2_ vdWHs is shown in Figure 3. TaS_2_ and MoS_2_ (WSe_2_) maintain their intrinsic band structure while forming heterostructures. The metal properties of the TaS_2_ monolayer and semiconductor properties of the MoS_2_ (WSe_2_) monolayer are well preserved. In metal/semiconductor contacts, it is important to determine whether ShC or OhC contacts [48] are formed, reducing the Schottky barrier and improving charge injection efficiency. The weight band structure in Figure 3a,b showed that TaS_2_/MoS_2_ and TaS_2_/WSe_2_ vdWHs all formed Schottky contacts, and we found that the bandgap values of PBE were 1.58/1.63eV, respectively. It is well known that the Schottky barrier heights (SBH) of the n-type and p-type are determined by the Schottky-Mott rule [49] as Φ_Bn_ = *E_CBM_* − *E_F_* and Φ_Bp_ = *E_F_* − *E_VBM_*, where the conduction band minimum (CBM), valence band maximum value (VBM), and Fermi level are defined by *E_CBM_*, *E_VBM_*_,_ and *E_F_*, respectively. In addition, to confirm the formation of the Schottky contacts in such heterostructures [48], we further plot the work functions of metallic TaS_2_, semiconducting MoS_2_(WSe_2_) monolayers, and their corresponding vdWHs, as displayed in Figure 4a,b. The n-ShC SBH of TaS_2_/MoS_2_ vdWHs was 0.5 eV, and the p-ShC SBH of TaS_2_/WSe_2_ vdWHs was 0.49 eV. Notably, the SBH of TaS_2_/MoS_2_(WSe_2_) vdWHs is very small, indicating that the MoS_2_(WSe_2_) material can be considered an efficient 2D metal contact with the TaS_2_ material.

**Figure 5:**
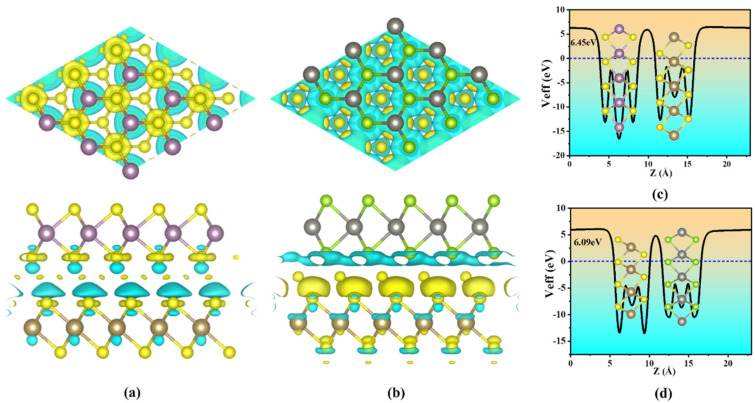
(**a**,**b**) In-plane average charge density difference of TaS_2_/MoS_2_, TaS_2_/WSe_2_ vdWHs, respectively. (**c**,**d**) In-plane average electrostatic potential of TaS_2_/MoS_2_, TaS_2_/WSe_2_ vdWHs, respectively. Inset represents the 3D charge density difference in the heterostructure. The yellow and cyan regions represent charge accumulation and depletion, respectively.

The charge density difference in TaS_2_/MoS_2_(WSe_2_) vdWHs is shown in Figure 5a,b. In order to further understand the charge distribution in TaS_2_/MoS_2_(WSe_2_) vdWHs, the electron density difference is calculated as follows [50,51]: Δ*ρ = ρ_vdWHs_ − ρ_TaS__2_ − ρ_MoS__2_(_ρWSe__2_)*. Here, *ρ_vdWHs_*, *ρ_TaS__2_*, and *ρ_MoS__2_* (*_ρWSe__2_*) represent the TaS_2_/MoS_2_(WSe_2_) combination vdWHs charge density and isolated TaS_2_ and MoS_2_(WSe_2_) monolayers, respectively.

The yellow area represents charge accumulation, whereas the cyan area represents charge depletion. Figure 5a,b clearly show that charge transfer occurs at the contact interface. For Figure 5a, the charge distribution is mainly concentrated at the contact interface between TaS_2_ and MoS_2_, where electrons are consumed in the Ta-S layer and accumulated in the Mo-S layer. As shown in Figure 5b, the charge distribution was mainly concentrated at the contact interface of TaS_2_ and WSe_2_, with electrons consumed on the W-Se layer and accumulated on the Ta-S layer. Therefore, the results indicate that the TaS_2_ and MoS_2_(WSe_2_) layers in the corresponding vdWHs exhibit weak interlayer interactions.

Figure 5c,d illustrate the mean in-plane average electrostatic potential of TaS_2_/MoS_2_(WSe_2_) vdWHs, respectively. Since the potential of TaS_2_ is higher than that of 2D MoS_2_, and the potential of 2D WSe_2_ is higher than that of TaS_2_, it indicates that the charge is transferred from the Ta-S layer to the Mo-S layer, and from the W-Se layer to the Ta-S layer, which is consistent with Figure 5a,b, which show that the direction of charge transfer is consistent. It can be seen that the interfacial charge transfer leads to the existence of a built-in electric field. Therefore, carrier mobility and charge injection may be affected. In addition, in order to prove that TaS_2_/MoS_2_(WSe_2_) vdWHs is suitable for high-performance nanodevices, it is essential to examine the carrier mobility of vdWHs. Therefore, carrier mobility and charge injection may be affected. In addition, in order to prove that TaS_2_/MoS_2_(WSe_2_) vdWHs are suitable for high-performance nanodevices, it is essential to examine the carrier mobility of vdWHs. For 2D systems, carrier mobility is closely related to effective mass because μ = eτ/m*. Thus, we established the effective masses of electrons (m_e_*) and holes (m_h_*) by fitting the band-edge dispersion VBM and CBM as follows:

$$\frac{1}{m^{*}}=\frac{1}{\hbar }\times \frac{\partial^{2}E\left(k\right)}{\partial k^{2}}$$

Here, ℏ is Planck’s constant and k is the wave vector. Our calculated m_e_* and m_h_* of TaS_2_/MoS_2_(WSe_2_) vdWHs are listed in Table 1. The effective mass values that can find these electrons and holes are very small, which proves that TaS_2_/MoS_2_(WSe_2_) vdWHs have a high carrier mobility. Therefore, they can be potential candidates for high-speed nanodevice applications.

### 2.3. Heterostructures under Interlayer Distance

**Figure 6:**
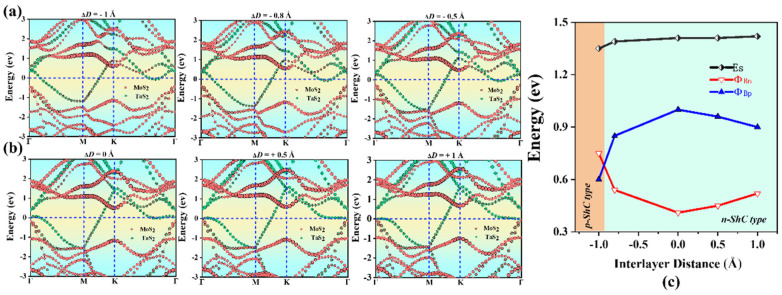
Projected band structures of TaS_2_/MoS_2_ vdWHs at different interlayer distances in (**a**,**b**). The MoS_2_ and TaS_2_ layers in (**a**) are separated by red and green circles, respectively. (**c**) Evolution of the contact barrier in the TaS_2_/MoS_2_ heterostructure at different interlayer distances.

**Figure 7:**
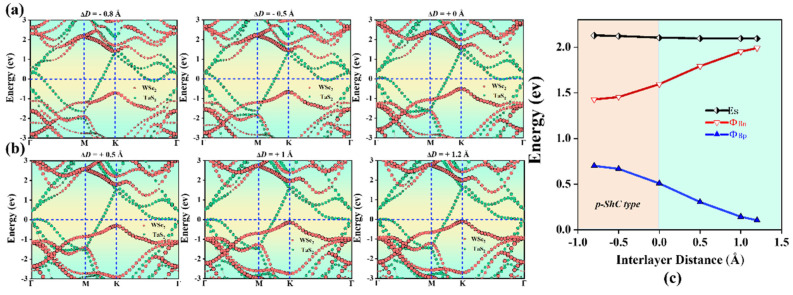
Projected band structures of TaS_2_/WSe_2_ vdWHs at different interlayer distances in (**a**,**b**). The WSe_2_ and TaS_2_ layers in (**a**) are separated by red and green circles, respectively. (**c**) Evolution of the contact barrier in the TaS_2_/WSe_2_ heterostructure at different interlayer distances.

It is well known that applying mechanical strain to change interlayer coupling can adjust the interface properties of heterostructures. Controllable SBH and contact types in TaS_2_/MoS_2_(WSe_2_) vdWHs are one of the most important challenges to improve the performance of nanodevices. Therefore, we further investigate the effect of strain engineering by adjusting the interlayer distance and applied electric field. Furthermore, it is worth noting that the interlayer distance in 2D-based vdWHs can be controlled by scanning tunneling microscopy [52] or vacuum thermal annealing [53]. Here, the strain is applied by adjusting the layer spacing, defined as ΔD = D − D_0_, where the original D of MoS_2_ and WSe_2_ is 3.0 Å and 2.9 Å, respectively, and D_0_ is the layer spacing after the strain. The tensile strain is defined by increasing the interlayer distance D, while the compressive strain is defined by decreasing D. ΔD < 0 represents the compressive strain, while ΔD > 0 represents the tensile strain. As shown in Figure 6a,b, for TaS_2_/MoS_2_ vdWHs, it is found that the tensile strain tends to increase Φ_Bn_ and decrease Φ_Bp_. In the case of ΔD > 0, the CBM of the MoS_2_ layer moves upward away from the Fermi level, resulting in the increase in Φ_Bn_. On the other hand, VBM moves upward towards the Fermi level, resulting in a decrease in Φ_Bp_. TaS_2_/MoS_2_ vdWHs changes with the SBH of ΔD, as shown in Figure 6c. When 0 < ΔD < 1 Å tensile strain, it can be seen that Φ_Bp_ > Φ_Bn_. In this case, TaS_2_/MoS_2_ has the n-ShC type. Moreover, TaS_2_/MoS_2_ still maintains the n-ShC type under −0.8 < ΔD < 0 Å compression strain. However, when ΔD ≤ −0.8 Å, it is observed in Figure 6c that Φ_Bn_ is gradually larger than Φ_Bp_, resulting in a transition from the n-ShC type to p-ShC type. Therefore, the SBH and contact types in TaS_2_/MoS_2_ vdWHs can be adjusted by changing the layer spacing. On the contrary, As shown in Figure 7a,b, for TaS_2_/WSe_2_ vdWHs, when ΔD is greater than −0.8 Å and less than 1.2 Å, according to the overall trend, we find that the WSe_2_ layer of the CBM moves upward away from the Fermi energy level, resulting in an increase in Φ_Bn_. On the other hand, the VBM moves upward towards the Fermi level, resulting in a decrease in Φ_Bp_. TaS_2_/WSe_2_ vdWHs changes with the SBH of ΔD, as shown in Figure 7c. It was observed that the SBH of TaS_2_/WSe_2_ vdWHs varied linearly with layer distance. When 0 < ΔD ≤ 1.2 Å tensile strain, it can be seen that Φ_Bn_ > Φ_Bp_. In this case, TaS_2_/WSe_2_ has a p-ShC type. In addition, when −0.8 ≤ ΔD < 0 Å compressive strain, Φ_Bn_ is still larger than Φ_Bp_. In this case, it indicates that TaS_2_/WSe_2_ still maintains the p-ShC type. Therefore, the SBH in TaS_2_/WSe_2_ vdWHs can be adjusted by changing the layer spacing, but the contact type cannot be adjusted.

### 2.4. Heterostructures under Electric Field

**Figure 8:**
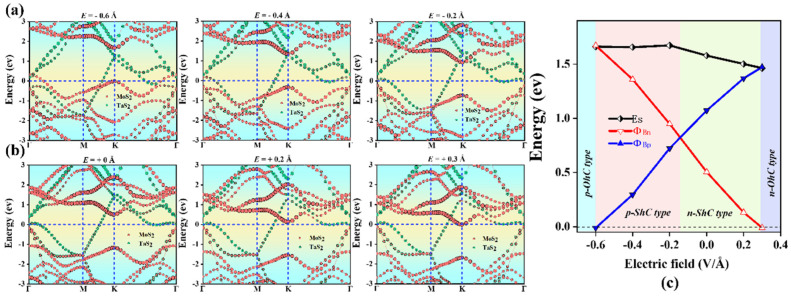
Projected band structures of TaS_2_/MoS_2_ vdWHs along the z direction under different electric fields applied in (**a**,**b**). The MoS_2_ and TaS_2_ layers in (**a**) are separated by red and green circles, respectively. (**c**) Evolution of the contact barrier in the TaS_2_/MoS_2_ heterostructure under different electric fields.

**Figure 9:**
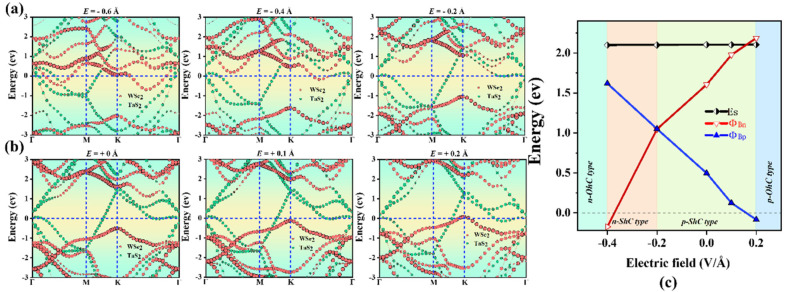
Projected band structures of TaS_2_/WSe_2_ vdWHs along the z direction under different electric fields applied in (**a**,**b**). The WSe_2_ and TaS_2_ layers in (**a**) are separated by red and green circles, respectively. (**c**) Evolution of the contact barrier in the TaS_2_/WSe_2_ heterostructure under different electric fields.

Furthermore, we considered the effect of the electric field on the electronic properties and contact types of TaS_2_/MoS_2_ (WSe_2_) vdWHs, as shown in Figure 8 and Figure 9. It can be observed that the SBH of TaS_2_/MoS_2_ (WSe_2_) vdWHs changes linearly with the electric field. Here, the applied electric field is applied in the z direction of vdWHs. As shown in Figure 8a,b, for TaS_2_/MoS_2_ vdWHs, by applying a positive electric field, the CBM of the MoS_2_ layer moves down to the Fermi level, resulting in a decrease in Φ_Bn_. Instead, VBM moves downward away from the Fermi level, causing Φ_Bp_ to increase. Interestingly, changes in SBH and contact types can be seen in Figure 8c. When a positive electric field of 0 < E < 0.3 V/Å is applied, it can be seen that Φ_Bp_ > Φ_Bn_. In this case, the n-ShC type exists for 1T-TaS_2_/2H-MoS_2_ vdWHs. Surprisingly, when a positive electric field of E ≥ 0.3 V/Å is applied, it is found that the CBM of MoS_2_ moves down through the Fermi level and can form a transition from the n-ShC type to n-OhC type in TaS_2_/MoS_2_ vdWHs. Similarly, when a negative electric field of −0.14 < E ≤ 0 Å is applied, it can be found that Φ_Bp_ > Φ_Bn_. In this case, the n-ShC type still exists for TaS_2_/MoS_2_ vdWHs. However, when a negative electric field of −0.6 < E ≤ −0.14 Å is applied, it can be observed that Φ_Bn_ is gradually larger than Φ_Bp_, indicating that TaS_2_/MoS_2_ vdWHs can form a transition from the n-ShC type to p-ShC type. In addition, when a negative electric field of E ≤ −0.6 V/Å is applied, it can be found from the figure that the VBM of MoS_2_ moves upward through the Fermi level, forming a transition from the p-ShC type to p-OhC type. Similarly, as shown in Figure 9a,b, for TaS_2_/WSe_2_ vdWHs, the CBM of the WSe_2_ layer moves upward away from the Fermi level by applying a positive electric field, resulting in an increase in Φ_Bn_. In contrast, VBM moves upward towards the Fermi level, resulting in a decrease in Φ_Bp_.

Interestingly, changes in SBH and contact types can be seen in Figure 9c. When a positive electric field of 0 < E < 0.2 Å is applied, it can be seen that Φ_Bn_ > Φ_Bp_. In this case, TaS_2_/WSe_2_ vdWHs will form the p-ShC type. Shockingly, when a positive electric field of E ≥ 0.2 Å was applied, the VBM of WSe_2_ was found to move upward through the Fermi level, forming a transition from the p-ShC type to p-OhC type. Furthermore, when a negative electric field −0.2 < E ≤ 0 Å is applied, it can be found that Φ_Bn_ > Φ_Bp_. In this case, there is the p-ShC type in TaS_2_/WSe_2_ vdWHs. However, when a negative electric field of −0.4 < E ≤ −0.2 Å is applied, it can be found that Φ_Bp_ > Φ_Bn_ can form a transition from the p-ShC type to p-OhC type. Moreover, when a negative electric field of E ≤ −0.4 Å is applied, the CBM of WSe_2_ can be found to move down through the Fermi level, resulting in a transition from the n-ShC type to n-OhC type in TaS_2_/WSe_2_ vdWHs. All the above results indicate that the application of an electric field can regulate the contact type and SBH of TaS_2_/MoS_2_ (WSe_2_) vdWHs, as well as the conversion of Schottky contact to ohmic contact from the n-ShC type to p-ShC type. Our results can provide a new approach for the design of future electron nanodevices based on metal/semiconductor TaS_2_/MoS_2_ (WSe_2_) vdWHs.

## 3. Computational Methods

Structural optimization and property calculations are performed within the density functional theory framework [54], as implemented in the Vienna ab initio simulation package [55] (VASP), where the ion−electron interaction is implemented by the projector-augmented plane wave (PAW) approach [56]. The structural models, volumetric data such as electron/nuclear densities, and crystal morphologies are processed by Visualization for Electronic Structural Analysis [57] (VESTA). The electronic exchange-correlation functional is treated using the generalized gradient approximation [58] (GGA) in the form proposed by Perdew, Burke, and Ernzerhof [59] (PBE). The energy cutoff of the plane waves is set to 350 eV, with an energy precision of 10^−6^ eV. Atomic positions are fully relaxed until the force on each atom is less than 10^−3^ eV/Å. The supercell method is considered to simulate the monolayer, where a vacuum distance of ∼20 Å is used to eliminate the interaction between the adjacent layers. Considering that the GGA usually underestimates the bandgaps, we adopt the Heyd−Scuseria−Ernzerhof (HSE06) hybrid functional [60] to calculate the band structures. The dynamic stabilities and phonon dispersion curves are computed with the supercell approach, as implemented in the Phonopy code [61]. The dipole correction was also included in the calculations.

## 4. Conclusions

In summary, we investigated the electronic structure and interfacial properties of metal/semiconductor 1T-TaS_2_/2H-MoS_2_(2H-WSe_2_) vdWHs by using first-principles calculations. The metallic character of the monolayer 1T-TaS_2_ and the intrinsic properties of the monolayer 2H-MoS_2_(2H-WSe_2_) semiconductor are preserved in TaS_2_/MoS_2_(WSe_2_) vdWHs. We demonstrate that TaS_2_/MoS_2_ and TaS_2_/WSe_2_ form n-ShC type and p-ShC type Schottky contacts, with ultralow Schottky barrier heights (SBH) of 0.51 eV and 0.49 eV. The results show that TaS_2_ can be considered as an effective metal contact with high charge injection efficiency for MoS_2_, WSe_2_ semiconductors. Furthermore, the electronic structure and interfacial properties of TaS_2_/MoS_2_ (WSe_2_) vdWHs are tunable under the action of the strain and electric field, which can not only induce the change in SBH, but also form a transition from the n-ShC type to p-ShC type and from ShC to ohmic contacts. Our findings suggest that metal/semiconductor TaS_2_/MoS_2_(WSe_2_) vdWHs are promising candidates for optoelectronic devices.

## Figures and Tables

**Table 1:** Calculated lattice parameters (*a*), interlayer distance (*D*), bandgap (*E_g_*) obtained by PBE calculations, and effective mass for electrons (*m**_e_^x^***) and holes (*m**_h_^y^***) along the x and y directions.

	*a* (Å)	*D* (Å)	*E_g_* (eV)	*m_e_^x^*/*m*_0_	*m_h_^y^*/*m*_0_	Contact Types
*2H-MoS_2_*	3.184	_	1.66	0.24	1.25	_-
*2H-WSe_2_*	3.184	_	1.46	0.42	0.49	_-
*TaS_2_/WSe_2_*	3.186	2.9	1.63	_-	_-	p-ShC
*TaS_2_/MoS_2_*	3.186	3.0	1.58	_-	_-	n-ShC

## Data Availability

Not applicable.

## Supplementary Materials

The following supporting information can be downloaded at: https://www.mdpi.com/article/10.3390/molecules28145607/s1. See the supplementary material for results of orientation-dependent Young’s modulus and Poisson’s ratio. Figure S1. Polar Plots of (a) Young’s modulus and (b) Poisson’s ratio of 1T-TaS_2_/2H-MoS_2_ vdWHs at the ground state. Polar Plots of (c) Young’s modulus and (d) Poisson’s ratio of 1T-TaS_2_/2H-WSe_2_ vdWHs at the ground state.

## Sample Availability

Samples of the compounds are available from the authors upon request.
