# Supplementary material for: Tunable Contact Types and Interfacial Electronic Properties in TaS2/MoS2 and TaS2/WSe2 Heterostructures
Source: Molecules. 2023 Jul 24;28(14):5607. doi: 10.3390/molecules28145607 (PMC10385421; doi:10.3390/molecules28145607)
Supplement: Supplementary file 1 [file molecules-28-05607-s001.zip › molecules-2468864-supplementary.pdf]

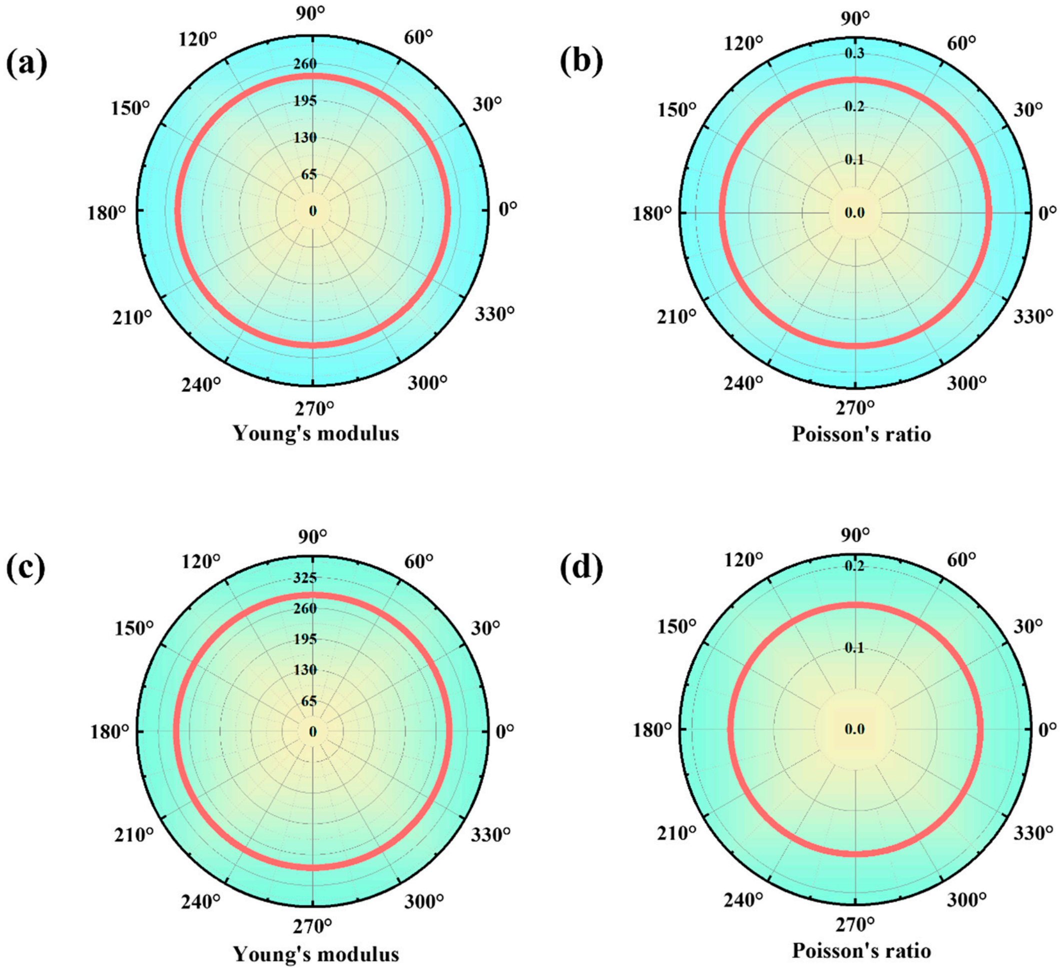

Figure S1. Polar Plots of (a) Young's modulus and (b) Poisson's ratio of 1T-TaS_2/2H-MoS_2 vdWHs at the ground state. Polar Plots of (c) Young's modulus and (d) Poisson's ratio of 1T-TaS_2/2H-WSe_2 vdWHs at the ground state.
